# Prediction of pre-eclampsia using gestosis score

**DOI:** 10.6026/9732063002001390

**Published:** 2024-10-31

**Authors:** Shamimah Ayazahmed Kharodia, Nitin S. Kshirsagar, Supriy Patil

**Affiliations:** 1 Department of Obstetrics and Gynecology, Krishna Institute of Medical Sciences, Karad - 415110, Maharashtra, India

**Keywords:** Pre-eclampsia, hypertensive pregnancy, gestosis score, hypertensive disease of pregnancy, antenatal visits, questionnaire

## Abstract

Pre-eclampsia (P-EP) is a hypertensive pregnancy (HPT-PG) condition that develops after 20 weeks of gestation, with or without proteinuria and is differentiated by vasospasm and vascular endothelial dysfunction. As a result, it is important to evaluate and predict P-EP using gestosis score (GT-S). We examined 229 pregnant patients at five prenatal visits and P-EP, using the Hypertensive Disease of Pregnancy (HDP) GT-S calculator app and a questionnaire that included 27 risk variables. We discovered a strong association between increased GT-S and the development of P-EP. As a result, GT-S is a reliable predictor of P-EP.

## Background:

Studies have shown that Pre-eclampsia (P-EP) is one of the most common PG complications, accounting for 4.6% of all PG worldwide [1, 2]. According to studies done in India, the total pooled prevalence of P-EP was 11% [3]. P-EP is responsible for a significant number of maternal and perinatal complications on a global scale. These complications can range from issues with the placenta, blood clotting (BC), fluid buildup (FB) in the lungs, kidney problems (KP), heart rhythm disturbances (HRD), and impacts on various organs like the liver, brain, and lungs. Additionally, P-EP can lead to problems with fetal growth, premature births, and even fetal deaths [[Bibr R04]]. Research have shown several maternal risk factors have been identified as being positively associated with the development of P-EP, including increased age, parity, comorbidities, family h/o, previous personal h/o, ethnicity, and investigative markers such as thyroid profile, Uterine Artery Doppler Velocimetry (UADV), PAPP-A levels, placental Insulin-like growth factor (IGF) levels, and specific systemic conditions [5]. Individual studies have established these elements, therefore, taking them all into account and devising a scoring system for P-EP prediction was crucial, particularly in locations with limited resources and a lack of biomarker testing capabilities. HPT illnesses complicate 5% to 10% of all PG, forming a fatal triad with hemorrhage and infection, and are believed to be responsible for 18% of maternal mortality globally. HPT diseases are the 2nd leading cause of maternal mortality in underdeveloped nations, including India [6]. It may also occur during the postpartum period (PP-P). P-EP is a condition with multiple etiologies that can lead to various severe complications, including EP, cerebral hemorrhage, CV problems, hepatic failure, acute renal failure, pulmonary edema, ARDS (acute respiratory distress syndrome), DIC (disseminated intravascular coagulation), hemolysis, elevated liver enzymes, and low platelets (HELLP) syndrome, and retinal detachment. P-EP is potentially linked to utero-placental insufficiency, which is the primary factor contributing to fetal abnormalities such as short for gestational age (GA), low birth weight (LBW), intrauterine fetal death (IUFD), and complications related to preterm delivery(PT-D) [7]. Therefore, it is of interest to report & evaluate the validity of GT-S in predicting P-EP & EP.

## Materials and Methods:

The current hospital-based prospective observational study was conducted in the department of Obstetrics and Gynecology, Krishna Vishwa Vidhyapeeth, Karad starting from June 2022 to November 2023 for around 1.5 years. 229 patients in total were screened & assessed using HDP GT-S calculator app as shown in Table 1. Here, mild, moderate and high risk factors were quantified as 1, 2 and 3 respectively. Score was calculated using questionnaire of 27 risk factors. This app was available on Android which is free, easy to use, has automatic mail generation within 1 minute and printout can be removed. When the total score was more than or equal to 3, the pregnant women is marked as 'At risk for HDP'. If the score </= 2 , the app itself says to look for the dynamic score when the patient visit the ANC clinic for the next time because variable factors like MAP (mean arterial pressure) and weight gain may alter the score during each visit.

## Inclusion criteria:

Pregnant women with 1^st^ visit before 20 weeks who were willing to continue their PG & willing to come for follow-up

## Exclusion criteria:

[1] Do not give informed consent.

[2] Lost to follow-up in subsequent visit.

[3] Those who were already on Tablet Aspirin.

## Statistical analysis:

Chi-square test or Fisher's exact test for categorical variables was used. Student's t-test and Mann- Whitney U test for continuous variables. For p value <0.05 was considered as statistically significant difference.

## Results:

Table 2 shows that, majority of women 212 (92.6%) are aged 19-35 years. Women younger than 19 years account for 12 (5.2%) patients, while those older than 35 years constitute 5 (2.2%) patients. Table 3 shows majority of women (59.8%) have a normal BMI (18.5 - 24.9 kg/m^2^). Data shows that OW-W (25-29.9 kg/m^2^) account for 23.1% of patients, while 7.4% are UN (BMI <18.5 kg/m^2^). Obesity is divided into two categories: 5.2% of patients have a BMI over 30 kg/m^2^ and 4.4% have a BMI over 35 kg/m^2^. Table 4 shows that, the majority of patients, 194 (84.7%), are MG, while 35 Patients (15.3%) are PGV. Table 5 shows that, out of 229 patients, the majority of patients, 176 (76.9%) have inter-pregnancy interval of 2-7 years. A smaller proportion, 41 Patients (17.9%) have intervals of less than 2 years, and 12 Patients (5.2%) have intervals greater than 7 years. Table 6 shows that, vast majority, 222 women (96.9%) have single foetus. A small proportion, 7 Patients (3.1%) are having multiple PG. Table 7 shows that, out of 229 women, 55 women (24.0%) were diagnosed with MA (< 11gm%), while 174 women (76.0%) did not meet the criteria for anemia (≥ 11gm%). Table 8 shows that, out of 229 Patients, 207 Patients (90.4%) received NT, 20 Patients (8.7%) underwent OID therapy, 2 Patients (0.9%) opted for ART (IVF/ICSI), and no Patients underwent ART (OD). Table 9 shows that, G-DM was present in 48 Patients (21.0%), HPT disease during previous PG in 60 Patients (26.2%), woman born as small for GA in 7 Patients (3.1%), short duration of PN (cohabitation) (<12 months) in 7 Patients (3.1%), MAP >85 mmHg in 70 Patients (30.6%), and excessive WG during PG in 1 case (0.4%).

Table 10 shows that, POS was present in 7 Patients (3.1%), C-VD (dyslipidemia) in 1 case (0.4%), pre-gestational DM in 19 Patients (8.3%), C-HPT in 15 Patients (6.6%), mental disorders (*e.g.,* schizophrenia) in 4 Patients (1.7%), inherited/acquired TBP in 1 case (0.4%), MT-C-KD in 2 Patients (0.9%), AI-D (SLE/APLA/RA) in 6 Patients (2.6%), and MT-HT in 60 Patients (26.2%). Table 11 shows that, there were 2 Patients (0.9%) with a family h/o of CVD and 45 Patients (19.7%) with a family h/o of PE. Table 12 shows that, maternal HT (Score 1) was the most prevalent risk factor, affecting 60 Patients (26.2%). Other notable risk factors include MAP >85 mmHg (Score 1) with 70 Patients (30.6%), G-DM (Score 2) affecting 48 Patients (21.0%), and HPT disease during previous PG (Score 2) with 60 Patients (26.2%). Table 13 shows that, a strong correlation between higher GT-S and the development of P-EP. This could indicate that higher GT-S was significant risk factor for P-EP. Table 14 shows that, among those who were prescribed aspirin, 36 Patients (61.0%) developed P-EP, while 9 cases (5.3%) did not. In contrast, among those not prescribed aspirin, 23 cases (39.0%) developed P-EP, with 161 cases (94.7%) not developing it. The results are statistically significant it means on aspirin had a notably higher percentage of P-EP cases compared to those who were not on aspirin. (P = <0.001).

## Discussion:

During the 20th week of PG, HPT issues may arise and have an impact on various bodily systems. Diagnostic criteria may include elevated albumin levels, pathological edema and BP readings reaching 140/90 mmHg [8]. There are several complications linked to PG-induced HPT, such as P-EP, EP, HELLP syndrome, hepatic and RF, retinal detachment, heart abnormalities and PE [9]. According to the short-term statistics from the National EP Registry (NER 2013), the prevalence rates of EP, P-EP, Federation of Obstetric and Gynaecological Societies of India (FOGSI) and International Federation of Obstetricians and Gynaecologists (ICOG) are 1.9% and 10.3%, respectively. About half of these cases are detected during the antepartum phase, while only 13% of patients are documented in the PP-P [10]. In research conducted by Iman *et al.* in 2023, the average age of the participants was 25.71±5.9 years, and the average gestational age was 11.9±2.19 weeks [11]. In their study, Mishra *et al.* examined the HDP gestosis score as a potential indicator of pregnancy-induced hypertension (PIH) in older individuals [12]. Our study revealed that the odds ratios for pre-eclampsia were 5.21 (95% confidence interval [CI] - 2.75 - 9.85) and 4.09 (95% CI - 2.05 - 8.18) for those aged over 35 and under 19 years old, respectively. Those who had a BMI more than 30 showed a statistically significant increased risk of P-EP (78.9%) in the study conducted by Manhar *et al.* [13] When the body mass index (BMI) increased, the study found that there was a corresponding rise in the number of instances of P-EP (p 0.0001) [13]. According to the findings of a study conducted by Iman *et al.* the bulk of the participants are first-time mothers, making up 66.72% of the total, while just 33.28% are MG [11]. In present study the majority of women 212 (92.6%) are aged 19-35 years. Women younger than 19 years account for 12 (5.2%) of patients, while those older than 35 years constitute 5 (2.2%) of patients. Most women (59.8%) have a normal BMI (18.5 - 24.9 kg/m^2^). Data also shows that OW-W (25-29.9 kg/m^2^) account for 23.1% of patients, while 7.4% are UN (BMI <18.5 kg/m^2^). Obesity is divided into two categories where 5.2% of patients have a BMI over 30 kg/m^2^ and 4.4% have a BMI over 35 kg/m^2^. Moreover, out of 229 patients, the majority of patients, 176 (76.9%), occur with an inter pregnancy interval of 2-7 years. A smaller proportion of 41 patients (17.9%), have intervals of less than 2 years, and 12 patients (5.2%) have intervals greater than 7 years. In present study the vast majority, 222 patients (96.9%), involve a single fetus. A small proportion of 7 patients (3.1%), are classified as multiple PG.

In the Manhar *et al.* study two patients (1.0%) with G-DM developed P-EP, compared to none without P-EP (p=0.001*). 70 patients (3.5%) with a history of HPT disorder in previous PG developed P-EP, significantly higher than those without P-EP (p=0.001*). Two patients (1.0%) with pre-G-DM developed P-EP, with none without P-EP (p=0.001*) [13]. Sravani *et al.* found PGV status was the most prevalent risk factor, observed in 14 patients (43.75%), indicating that 1st time PG are strongly linked to P-EP. Age over 35 years was noted in 4 patients (12.5%), suggesting advanced maternal age as another significant risk factor. HT, C-HPT, HPT in previous PG, and G-DM each accounted for 3 (9.375%), 2 (6.25%), 2 (6.25%) and 2 (6.25%) cases, respectively, further highlighting their associations with P-EP [14]. In present study there were 2 patients (0.9%) with a family history of CVD and 45 patients (19.7%) with a family h/o of PE. In present study MT-HT (Score 1) was the most prevalent risk factor, affecting 60 patients (26.2%). Other notable risk factors include MAP >85 mmHg (Score 1) with 70 patients (30.6%), G-DM (Score 2) affecting 48 patients (21.0%), and HPT disease during previous PG (Score 2) with 60 patients (26.2%). In the Manhar *et al.* study the sensitivity of the score is 50%, indicating that it correctly identifies half of the cases that develop P-EP [13].

Specificity is high at 96.43%, indicating a low rate of false positives. The positive predictive value (PPV) is 72.73%. The negative predictive value (NPV) is 91.01%, indicating that the score correctly identifies a vast majority of cases that do not develop pre-eclampsia. The area under the curve (AUC) is 0.95, demonstrating strong overall predictive accuracy of the GT-S ≥3 for P-EP. Wang *et al.* conducted a meta-analysis with total of 39 articles, including 29 studies with high- risk pregnant women and 10 with the general population, aspirin showed a 28% reduction in preeclampsia (PE) incidence (RR 0.72, 95% CI 0.62-0.83) in high-risk groups and 30% (RR 0.70, 95% CI 0.52-0.95) in the general population. Subgroup analyses indicated 75 mg/day dosage significantly reduced PE risk (RR 0.50, 95% CI 0.32-0.78), with earlier initiation (12-16 weeks) showing stronger efficacy (RR 0.62, 95% CI 0.53-0.74) [15]. Almasi-Hashiani *et al.* conducted a systematic review and meta-analysis to assess the risk of preeclampsia (PE) among women who conceived using assisted reproductive technology (ART). They analyzed data from 156,246 ART cases (including 14,560 PE cases) and 6,558,249 non-ART cases (with 202,064 PE cases) across 48 primary studies. The meta-analysis revealed a significant association, indicating a 1.708-fold higher risk of PE in the ART group compared to the non-ART group (95% CI = 1.111- 2.624, p = 0.015) [16]. If the gestosis score was 3 or higher, it was 97.03% likely that PE would happen, 97.51% of the time it was accurate, 85.51% of the time it was sensitive, and 83.51% of the time it was positive. Overall, it seems to be a unique early marker with diagnostic accuracy of 95.35% for prediction of the development of PE, allowing for prompt care of the patients and allowing them to reduce the negative outcomes [17]. Gestational hypertension poses significant risks to pregnancy outcomes. This condition must be diagnosed and managed, but also strategies must be developed to identify pregnant women at risk of gestational hypertension. The researchers came to the conclusion that the gestosis score is a good way to screen women in health centers that can't do ultrasonography or other expensive biochemical tests [18].

## Conclusion:

Patients with a GT-S of 3 or more showed a progressive increase in P-EP severity across subsequent visits. Data shows the importance of regular monitoring using the GT-S to predict and manage P-EP effectively, with aspirin use showing a significant reduction in P-EP incidence.

## Figures and Tables

**Table 1:** GT-S (Gestosis score)

**Risk factor included in Gestosis score**	**Score**
Woman born as small for gestational age	1
Maternal anemia	1
Age older than 35 years	1
Age younger than 19 years	1
Obesity (BMI > 30)	1
Nulligravida	1
Short duration of paternity (cohabitation)	1
Family history of PE	1
Family history (H/O) of cardiovascular disease	1
Polycystic ovary syndrome	1
Interpregnancy interval of more than 7 years	1
Assisted reproductive (IVF/ICSI) Treatment	1
Maternal hypothyroidism	1
Chronic vascular disease (dyslipidaemia)	1
Excessive weight gain during pregnancy	1
MAP > 85 mmHg	1
Gestational diabetes mellitus	2
Obesity (BMI > 35)	2
Multiple pregnancy	2
Hypertensive disease during previous pregnancy	2
Pre-gestational diabetes mellitus	3
Chronic hypertension	3
Mental disorders (e.g., schizophrenia)	3
Inherited/acquired thrombophilia	3
Maternal chronic kidney disease	3
Autoimmune disease (SLE/APLA/RA)	3
Assisted reproductive (Ovum Donor or surrogacy) treatment	3

**Table 2:** Age of women

**Age of woman**	**Patients**	**Percentage**
< 19 years	12	5.20%
19-35	212	92.60%
> 35 years	5	2.20%
Total	229	100.00%

**Table 3:** BMI of women

**BMI of woman**	**Patients**	**Percentage**
Undernourished (UN)(< 18.5kg/m2)	17	7.40%
Normal (18.5 - 24.9 kg/m2)	137	59.80%
Overweight(OW) (25-29.9 kg/m2)	53	23.10%
Obesity (> 30 kg/m2)	12	5.20%
Obesity (> 35 kg/m2)	10	4.40%
Total	229	100.00%

**Table 4:** Gravidity distribution

**Parity**	**Patients**	**Percentage**
Primigravida(PGV)	35	15.30%
Multigravida(MG)	194	84.70%
Total	229	100.00%

**Table 5:** I-PG-I

**Interpregnancy interval (I-PG-I)**	**Patients**	**Percentage**
< 2 year	41	17.90%
2-7 year	176	76.90%
> 7 year	12	5.20%
Total	229	100.00%

**Table 6:** No of foetus

**No. of foetus in current pregnancy**	**Patients**	**Percentage**
Single	222	96.90%
Multiple pregnancy	7	3.10%
Total	229	100.00%

**Table 7:** MA

**Maternal anemia (MA)**	**Patients**	**Percentage**
Yes (< 11gm %)	55	24.00%
No (> 11gm %)	174	76.00%
Total	229	100.00%

**Table 8:** Treatment - PGT

**Treatment for being pregnant(PGT)**	**Patients**	**Percentage**
No treatment(NT)	207	90.40%
Ovulation induction drug(OID)	20	8.70%
Assisted reproductive (IVF/ICSI) Treatment(ART)	2	0.90%
Assisted reproductive (OD) treatment	0	0.00%
Total	229	100.00%

**Table 9:** R/F

**Risk factors**	**Patients**	**Percentage**
Gestational diabetes mellitus (G-DM)	48	21.00%
Hypertensive(HPT) disease during previous PG	60	26.20%
Woman born as small for GA	7	3.10%
Short Duration of paternity(PN) (cohabitation) (< 12 month)	7	3.10%
Mean Arterial Pressure (MAP) >85 mmHg	70	30.60%
Excessive weight gain(WG) during PG	1	0.40%

**Table 10:** R/F co-morbidities

**Risk factors - Co-morbidities**	**Patients**	**Percentage**
Polycystic ovary syndrome (POS)	7	3.10%
Chronic vascular disease(C-VD) (dyslipidemia)	1	0.40%
Diabetes mellitus (DM) - Pre-gestational	19	8.30%
Chronic hypertension(C- HPT)	15	6.60%
Mental disorders (e.g., schizophrenia)	4	1.70%
Inherited/acquired thrombophilia (TBP)	1	0.40%
Maternal chronic kidney disease (MT-C-KD)	2	0.90%
Autoimmune disease(AI-D) (SLE/APLAS/RA)	6	2.60%
Maternal hypothyroidism(MT-HT)	60	26.20%

**Table 11:** R/F - Family h/o

**Risk factor - Family history**	**Patients**	**Percentage**
Family history (H/o) of cardiovascular disease(CVD)	2	0.90%
Family history of pulmonary embolism (PE)	45	19.70%

**Table 12:** R/F in GT-S

**Risk factor included in Gestosis score**	**Score**	**Patients**	**Percentage**
Woman born as small for gestational age	1	7	3.10%
Maternal anemia	1	55	24.00%
Age older than 35 years	1	5	2.20%
Age younger than 19 years	1	12	5.20%
Obesity (BMI > 30)	1	12	5.20%
Nulligravida	1	35	15.30%
Short duration of paternity (cohabitation)	1	30	13.10%
Family history of PE	1	45	19.70%
Family history (H/O) of cardiovascular disease	1	2	0.90%
Polycystic ovary syndrome	1	7	3.10%
Interpregnancy interval of more than 7 years	1	12	5.20%
Assisted reproductive (IVF/ICSI) Treatment	1	2	0.90%
Maternal hypothyroidism	1	60	26.20%
Chronic vascular disease (dyslipidemia)	1	1	0.40%
Excessive weight gain during pregnancy	1	1	0.40%
MAP > 85	1	70	30.60%
Gestational diabetes mellitus	2	48	21.00%
Obesity (BMI > 35)	2	10	4.40%
Multiple pregnancy	2	7	3.10%
Hypertensive disease during previous pregnancy	2	60	26.20%
Pre-gestational diabetes mellitus	3	19	8.30%
Chronic hypertension	3	15	6.60%
Mental disorders (e.g., schizophrenia)	3	4	1.70%
Inherited/acquired thrombophilia	3	1	0.40%
Maternal chronic kidney disease	3	2	0.90%
Autoimmune disease (SLE/APLA/RA)	3	6	2.60%
Assisted reproductive (Ovum Donor) treatment	3	0	0.00%

**Table 13:** GT-S various ANC visit & P-EP

**Gestosis Score**	**Antenatal Visits**					**Preeclampsia**	
	**First Visit (21-24 wks)**	**Second Visit (25-28 wks)**	**Third Visit (29-32 wks)**	**Forth Visit (33-36 wks)**	**Fifth Visit (37-40wks)**	**Developed (%)**	**Not developed (%)**
1	114	114	110	103	92	3 (3.3%)	89 (96.7%)
2	70	70	73	79	86	6 (7%)	80 (93%)
3	32	30	26	24	26	25 (96.2%)	1 (3.8%)
05-Apr	11	11	15	16	14	14 (100%)	0 (0%)
07-Jun	2	4	5	6	8	8 (100%)	0 (0%)
09-Aug	0	0	0	1	2	2 (100%)	0 (0%)
> 9	0	0	0	0	1	1 (100%)	0 (0%)
Total	229	229	229	229	229	59 (25.8%)	17 4.2%)

**Table 14:** Aspirin medication & P-EP

**Aspirin Medication**			**Preeclampsia**				
	**Developed**	**%**	**Not developed**	**%**	**Total**	**%**	**P value**
On Aspirin	36	61.00%	9	5.30%	45	19.70%	< 0.001
Not on Aspirin	23	39.00%	161	94.70%	184	80.30%	
Total	59	100.00%	170	100.00%	229	100.00%	
